# Editorial: Use of neuroimaging techniques for the prevention, assessment, and treatment of mood disorders

**DOI:** 10.3389/fpsyt.2022.1091676

**Published:** 2023-01-04

**Authors:** Gaia Romana Pellicano, Katie Aafjes-van Doorn, Alessandra Anzolin, Danilo Arnone, Gianluca Borghini

**Affiliations:** ^1^Department of Dynamic and Clinical Psychology, and Health Studies, Sapienza University, Rome, Italy; ^2^Ferkauf Graduate School of Psychology, Yeshiva University, New York, NY, United States; ^3^Department of Physical Medicine and Rehabilitation, Harvard Medical School, Spaulding Rehabilitation Hospital, Boston, MA, United States; ^4^Department of Psychiatry and Behavioral Science, College of Medicine and Health Sciences, United Arab Emirates University, Al Ain, United Arab Emirates; ^5^Department of Psychological Medicine, Centre for Affective Disorders, Institute of Psychiatry, Psychology and Neuroscience, King's College London, London, United Kingdom; ^6^Department of Molecular Medicine, Sapienza University of Rome, Rome, Italy

**Keywords:** mood disorder, neuroimage, ERP, ECG, treatment, fMRI

Mood disorders are common psychiatric conditions characterized by altered mood, energy levels, and an array of biological symptoms which also affects cognitive functions. The most common types of mood disorders are major depression, dysthymia (dysthymic disorder), bipolar disorder, mood disorder due to a general medical condition, and substance-induced mood disorder. Major depression is one of the most prevalent mood disorders that significantly contributes to the overall global disease burden (1). Over the years, neuroimaging studies have highlighted different aspects of mood disorders, from their neuroanatomy (2) to the brain functional activity during emotional information processing, and aberrant motivational and affective processing (3). Both structural and functional neuroimaging studies in mood disorders point toward the involvement of frontal brain regions, particularly in the ventromedial, ventrolateral, and orbitofrontal areas and the limbic system (2, 4, 5). Currently, neuroimaging findings have been also implemented in clinical practice to treat severe forms of depression (6). For instance, several neuromodulation techniques, including deep brain stimulation (DBS) (7), repetitive transcranial magnetic stimulation (rTMS) (8), and magnetic seizure therapy (9), are shown to alleviate depressive symptoms and improve quality of life of the patients.

Despite the recent progress in the field, syndromic identification and differentiation based on neuroimaging is not possible currently (4, 10), largely due to the heterogeneity of these disorders and, at the neurological level, the small magnitude of brain changes, and the lack of specificity (4, 5). Therefore, it is of pivotal importance to refine neuroimaging techniques in conjunction with better defined etiological models at brain level.

The empirical studies included in this Research Topic are specifically focused on major depression, aiming at a better understanding of the pathophysiology of this disease, through the use of the electroencephalogram (EEG) and the functional magnetic resonance. Here, we provide a summary of the studies presented in the Research Topic, contextualizing some of their main findings.

Cognitive dysfunctions (i.e., impairments in verbal and visual short and long-term memory, executive functions, psychomotor skills, and attention) in depression can affect several domains, persisting after remission, and are a target for new treatment strategies (11, 12). Event-related potentials (ERP) have been reported as a reliable method for investigating executive functions in depression because of their high temporal resolution. Sun et al. compared the neuroelectrical response in patients with major depression and healthy controls during a visual task that measures interference and conflict resolution (i.e., whether the participant believes that the conflict has been resolved, or the main issues addressed). The authors showed that depressed patients had a deficit in conflict control (i.e., abnormal cognitive conflict resolution), as reflected by reduced amplitudes of the lateralized readiness potential. Additionally, Jin et al. showed a significantly lower amplitude in the late positive potential during a high-order difficult task, associated with difficulty in suppressing non-relevant information in depressed patients compared to healthy controls. Yoon et al. presented the ERP results obtained on a large population of patients with suicidal attempts and suicidal ideation during an inhibitory control task. ERP results reflected a higher dysfunction in controlling inhibition in the suicide attempters compared to patients with suicidal ideation. Despite the low spatial resolution of the ERP technique, these electrophysiological changes detected in high temporal resolution may help to further explain the neural mechanisms responsible for cognitive impairments in depression and inform more personalized treatment approaches by using portable and cost-effective devices.

Moving toward higher spatial resolution techniques, neuroimaging studies have reported altered brain functions in major depression in specific cortical areas (13, 14) including the prefrontal cortex and the anterior cingulate cortex (15), while functional connectivity research suggested the involvement of the brainstem and other subcortical areas (16).

Using graph theory analysis, an effective method to describe modular segregation of brain networks, Lan et al. reported decreased segregation and increased integration between fronto-parietal network, cerebellum, and cingulo-opercular network. Notably, enhanced inter-module connectivity was significantly correlated with depressive symptoms. These findings might help underpin the relationship between brain network alterations and clinical manifestation of depression.

Converging evidence suggests that altered resting-state functional connectivity (RSFC) in the nucleus accumbens (NAc) is involved in the reward system in patients with depressive disorders (17, 18). In this context, Zhou et al. showed reduced static and dynamic RSFC between NAc and cortical and subcortical regions, which was positively correlated with depression severity. Jung et al. also found significantly lower RSFC between the cerebellum and the habenula (a structure that integrates inputs from the limbic system and basal ganglia) in major depression. A subsequent region of interest (ROI)-to-ROI analysis also indicated widespread reduction in RSFC in numerous subcortical areas. These new findings with static and dynamic functional connectivity could provide new complementary evidence of the critical role of the subcortical areas in depressive disorders.

Finally, Zeng et al. proposed an electroencephalographic (EEG)-based toolbox for brain functional connectivity analysis and network visualization. This new technological development promotes accessibility to methods focused on identifying biomarkers in depression and other psychiatric conditions. Furthermore, this toolbox effectively detects brain connectivity changes over time while facilitating the understanding and interpretation of electrophysiological information within a more friendly human-computer visualization framework.

The studies included in this topic were mainly focused on major depressive disorder and they can be considered proof-of-concept providing some important leads for further research in the field of major depression and other mood disorders. Mood disorders are complex and heterogeneous conditions with several clinical and non-clinical variables capable of influencing brain structure and function, such as comorbidities, trauma history, attachment style, cultural and societal milieu.

The adaptation of neuroimaging techniques in the clinical practice in this field is challenging (19). More research is necessary as the existing tools are lacking sensitivity and specificity and to date, there are not established imaging-based biomarkers for mood disorders diagnosis, as well as for other psychiatric diseases (20). Moreover, neuroanatomical alterations tend to be subtle and widespread, making difficult the discrimination between normal and pathological heterogeneity (21, 22). In parallel, treatments effects require evaluation to establish potential “normalization” of trajectories in relation to brain pathology.

Although the diagnostic utility of neuroimaging in psychiatry is currently limited, as technology develops, future neuroimaging-based methods are likely to emerge (23, 24) overcoming wearability, usability, and costs issues. For example, conventional EEG sensors require the injection of some conductive gel between scalp and electrodes, which is time consuming and may cause discomfort to the users. Recently, many EEG systems replaced standard nets with water-based electrodes with significant practical benefits (25). These new tools should be considered in light of cost-effectiveness since self-report questionnaires can be administered at no cost with high level of sensitivity and specificity, whilst neuroimaging is primarily used to rule out or confirm organic disorders. A significant change is expected in the future when neuroimaging-based techniques might contribute to objectively identify mood disorders, evaluate treatment responses, and convey alternative treatments as in the case of neurofeedback (5, 21, 23). Standardized protocols will then be required to guide diagnostic and interventional procedures in mood disorders. The present Research Topic, contributes to move toward this direction providing novel results from multi-modal studies, targeting different patients population with mood disorders.

## Author contributions

GRP and GB conceived and wrote the editorial. KA, AA, and DA wrote the editorial and supervised the work. All authors read, performed critical revisions, and approved the final version of the editorial.
